# Assessing the role of redox partners in *Tth*LPMO9G and its mutants: focus on H_2_O_2_ production and interaction with cellulose

**DOI:** 10.1186/s13068-024-02463-y

**Published:** 2024-02-01

**Authors:** Koar Chorozian, Anthi Karnaouri, Nefeli Georgaki-Kondyli, Antonis Karantonis, Evangelos Topakas

**Affiliations:** 1 Industrial Biotechnology & Biocatalysis Group, School of Chemical Engineering, National Technical University of Athens, Zografou Campus, 15772 Athens, Greece; 2 Laboratory of Physical Chemistry and Applied Electrochemistry, School of Chemical Engineering, National Technical University of Athens, Zografou Campus, 15772 Athens, Greece; 3 Laboratory of General and Agricultural Microbiology, Department of Crop Science, Agricultural University of Athens, 11855 Athens, Greece

**Keywords:** Lytic polysaccharide monooxygenase, *Tth*LPMO9G, Cellulose oxidation, Electron donors, H_2_O_2_, Catalase

## Abstract

**Background:**

The field of enzymology has been profoundly transformed by the discovery of lytic polysaccharide monooxygenases (LPMOs). LPMOs hold a unique role in the natural breakdown of recalcitrant polymers like cellulose and chitin. They are characterized by a “histidine brace” in their active site, known to operate via an O_2_/H_2_O_2_ mechanism and require an electron source for catalytic activity. Although significant research has been conducted in the field, the relationship between these enzymes, their electron donors, and H_2_O_2_ production remains complex and multifaceted.

**Results:**

This study examines *Tth*LPMO9G activity, focusing on its interactions with various electron donors, H_2_O_2_, and cellulose substrate interactions. Moreover, the introduction of catalase effectively eliminates H_2_O_2_ interference, enabling an accurate evaluation of each donor’s efficacy based on electron delivery to the LPMO active site. The introduction of catalase enhances *Tth*LPMO9G’s catalytic efficiency, leading to increased cellulose oxidation. The current study provides deeper insights into specific point mutations, illuminating the crucial role of the second coordination sphere histidine at position 140. Significantly, the H140A mutation not only impacted the enzyme’s ability to oxidize cellulose, but also altered its interaction with H_2_O_2_. This change was manifested in the observed decrease in both oxidase and peroxidase activities. Furthermore, the S28A substitution, selected for potential engagement within the His1–electron donor–cellulose interaction triad, displayed electron donor-dependent alterations in cellulose product patterns.

**Conclusion:**

The interaction of an LPMO with H_2_O_2_, electron donors, and cellulose substrate, alongside the impact of catalase, offers deep insights into the intricate interactions occurring at the molecular level within the enzyme. Through rational alterations and substitutions that affect both the first and second coordination spheres of the active site, this study illuminates the enzyme’s function. These insights enhance our understanding of the enzyme’s mechanisms, providing valuable guidance for future research and potential applications in enzymology and biochemistry.

**Supplementary Information:**

The online version contains supplementary material available at 10.1186/s13068-024-02463-y.

## Background

LPMOs have given a new perspective on how nature breaks down polymers like cellulose and chitin among other natural polysaccharides. Unlike hydrolytic enzymes that interact with individual fiber chains, LPMOs can break down polymeric chains even when they are still packed tightly [1]. LPMOs activate glycosidic bonds by hydroxylating the polysaccharide substrate at either the C1 [2] or the C4 [3] position. Activation of C–H and cleavage of glycosidic bonds in these crystalline polysaccharides necessitate powerful redox chemistry to surmount an activation energy barrier of approximately 95 kcal/mol [4, 5]. LPMOs are currently classified in eight Auxiliary Activity families (AA9-11,13-17) in the Carbohydrate Active enZyme database [6].

LPMOs are monocopper enzymes in which copper is coordinated by a conserved histidine brace [7]. Gaining insights into how the LPMO active site environment impacts the reactivity of the copper site might shed light on tailoring synthetic copper sites using a strategic design approach. Closing the knowledge gap between natural enzymatic processes and synthetic catalysts has the potential to increase the array of substrates suitable for efficient C–H bond activation and achieve higher turnover numbers observed in natural LPMOs [8, 9]. While the first coordination sphere involves the direct interactions of amino acids with the central metal atom, defining the active site, the second coordination sphere comprises a conserved hydrogen-bonding (H-bonding) network [10]. These are amino acids that, while not directly connected to the metal ion, play pivotal roles in maintaining the structural integrity of the active site, influencing the geometry of the ligands in the first sphere, and are often involved in proton transfers during catalysis. H-bond network is not necessarily the same across LPMO families, it is sometimes conserved within a given LPMO family, and usually rather conserved within clades of a given family. Engineering LPMOs through point mutations, coupled with computational and structural studies, provide invaluable data, however mutations to the second coordination sphere remain relatively unexplored. It is expected that these substitutions will alter to some extent enzyme activity and/or enzyme binding to its solid substrate [11–15].

The polysaccharide chain breakage by LPMO is driven by a O_2_ or H_2_O_2_ co-substrate mechanism [16]. Even though debates regarding the nature of the co-substrate persist, it is commonly accepted that LPMOs oxidize glycosidic bonds through a reactive Cu(II)–O^•−^ species [17]. The catalytic process commences with an electron donor reducing the solvent-exposed copper ion (Cu^2+^), generating the reactive Cu^+^ species. This Cu^+^ subsequently interacts with the substrate, facilitated by an oxygenated co-substrate. LPMOs will operate independently when incubated with an appropriate electron donor such as ascorbic acid, and O_2_ in the absence of their polysaccharidic substrate and produce H_2_O_2_ [16, 18]. Additionally, the potential for high concentrations of H_2_O_2_ to inactivate the enzyme introduces another layer of complexity [19–21]. Thus, when examining LPMOs, it is imperative to assess their relationship with H_2_O_2_ [16, 22] Regardless of the co-substrate used by LPMOs, an electron source is necessary to reduce the active site copper and trigger the oxidative reaction that these enzymes catalyze. A variety of electron donors have been shown to effectively drive the LPMO reaction. These include small-molecule redox partners [1, 16] other redox enzymes [23] as well as light-absorbing compounds originating from either biotic or abiotic sources [24, 25]. Recent studies have necessitated a re-evaluation of previous assumptions concerning the electron donors’ role in enzyme assays, with a particular emphasis on the potential role of H_2_O_2_ in LPMO reactions. It has been observed that many redox partners, over time, undergo oxidation and simultaneously generate H_2_O_2_ through the reduction of O_2_ [26]. Systematic studies investigating the effects of electron donors on LPMO activity are limited, and the reported activities of LPMOs in electron donors-driven reactions display substantial variation [27]. Factors contributing to this variation include differences in experimental setups, such as the selected pH, management of free copper, and consideration of the auto-oxidation of the electron donors [28, 29]. In addition to the roles of electron donors previously discussed, it is important to note that LPMOs require a ‘priming’ reduction in H_2_O_2_-dependent reactions to initiate the reaction, as first demonstrated by Bissaro et al. [16]. After this initial activation, LPMOs can continue multiple turnovers without further electron supply. On the other hand, monooxygenase activity requires a reduction step for each turnover as well as 2nd electron during the reaction. Furthermore, there is a possibility that H_2_O_2_ is generated in situ at the (reduced) active site, acting as a co-substrate and contributing to the observed monooxygenase activity, particularly in fungal LPMOs [26].

In this study, we examine the effects of various electron donors on *Tth*LPMO9G previously characterized [30] with a strong C1 regioselectivity, and a carbohydrate-binding module of Family 1. *Tth*LPMO9G activity was studied during oxidation of cellulose and explore the role of catalase in these reactions. By introducing catalase, we aim to minimize interference from H_2_O_2_, produced by the redox partners, to accurately assess each donor’s ability to provide electrons to the LPMO. The addition of catalase in an enzymatic mixture has been shown to boost the efficacy of LPMO, as previously described [31]. This approach allows for a clearer understanding of the efficacy of each electron donor without the interference of H_2_O_2_ produced by the donors. To deepen our understanding, this paper examines interactions within the amino acid environment of the active site’s first and second coordination spheres through rational design substitutions of *Tth*LPMO9G and its specific mutations, namely H140A and S28A. This approach facilitates an evaluation of their impact on oxidase, peroxidase, and LPMO monooxygenase/peroxygenase activities. Insights derived from this study not only illuminate novel associations among the enzyme, electron donors, H_2_O_2_, substrate, and product patterns but also offer valuable theoretical perspectives. These insights significantly enhance our understanding of LPMO, especially within the distinctive His1-Ser28 environment and the second-sphere histidine.

## Results and discussion

### Assessing the effect of various electron donors on *Tth*LPMO9G activity upon absence or presence of catalase

**Fig. 1:**
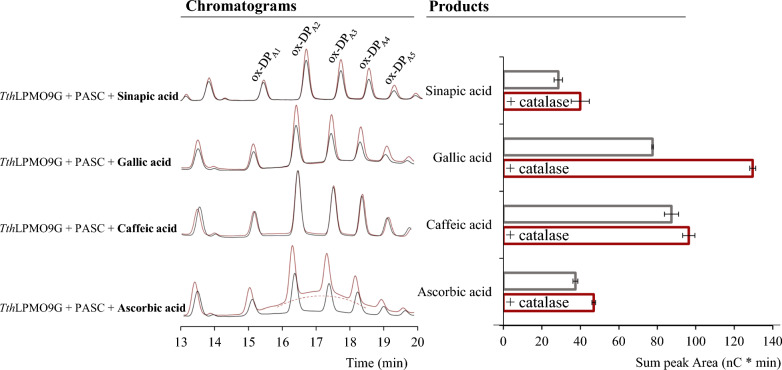
Α comparative analysis of total product release (in nC * min) resulting from cellulose oxidation by *Tth*LPMO9G (grey), following a 16-h reaction period. The same reaction was also conducted in the presence of catalase (red). Each reaction incorporated a 0.1% w/v PASC concentration, 4 μΜ *Tth*LPMO9G, and 1 mM electron donor, and analyzed via HPAEC-PAD. All experiments were carried out in 50 mM sodium acetate buffer, pH 6.0, at 45 °C. The chromatograms on the left delineate all eluted products for the 13–19 min retention window. Bars denote mean values, with error bars indicating the standard error derived from two independent experiments, each performed at least twice

The enzymatic activity of WT *Tth*LPMO9G was investigated on a model cellulosic substrate, specifically phosphoric acid-swollen cellulose (PASC; 0.1% w/v), under apparent monooxygenase conditions. The reactions initiated with various redox partners of 1 mM concentration and the LPMO-derived products were analyzed by high-performance anion-exchange chromatography with pulsed amperometric detection (HPAEC-PAD), where chromatographic peak areas were quantified at a retention time of 13–19 min, correlating to the elution of C1-oxidized products. In the results shown in Fig. 1, ascorbic acid, gallic acid, caffeic acid, and sinapic acid were assessed for their potential to reduce *Tth*LPMO9G. When the release of oxidized products from PASC was tested, caffeic acid exhibited the highest efficacy, followed sequentially by gallic acid, ascorbic acid, and sinapic acid. Some studies show that ascorbic acid appears to be a less potent electron donor for reactions with cellulose and LPMO than gallic and caffeic acids [29], while others argue that ascorbic acid is a more effective electron donor than gallic acid [23, 32]. However, the efficiency can differ based on the particular LPMO enzyme under study, as well as variations in reaction parameters like enzyme loading, substrate loading, and the concentration of the electron donor. Earlier studies have identified caffeic acid as a promising electron donor for LPMO, comparable in efficacy to ascorbic acid [33]. However, in our study it was observed that caffeic acid outperforms other electron donors, which was witnessed for the first time. This performance varies depending on the LPMO being characterized under specific reaction conditions.

Previous research has indicated that many redox partners will themselves be oxidized over time and can concomitantly produce H_2_O_2_ via the reduction of O_2_ [29]. In this study, the potential of various electron donors to act as electron donors was compared, specifically under the conditions applied for *Tth*LPMO9G with PASC. The goal was the comparative analysis of electron donors in reactions between LPMO and cellulose, with a particular focus on identifying the most efficient donor. However, electron donor-mediated production of H_2_O_2_ can interfere with LPMO activity in two distinct pathways: firstly, H_2_O_2_ can serve as a co-substrate for the LPMO peroxygenase reactions with PASC [16]. In addition, at higher concentrations, H_2_O_2_ might result in LPMO enzyme inactivation [16, 19–21]. Our goal was to exclude the interference from H_2_O_2_ produced by electron donors, thereby ensuring that the observed efficacy of electron donors is solely based on their ability to deliver electrons to the LPMO active site without the confounding influence of H_2_O_2_. This approach allows for a more accurate evaluation of electron donor efficiency in facilitating LPMO reactions with cellulose. To specifically mitigate the potential interference of H_2_O_2_, catalase, a known H_2_O_2_ scavenger, was incorporated into the reaction mixtures, so that it could remove the H_2_O_2_ produced by the redox partners. In this experimental design, conditions were maintained consistent with those for LPMO with PASC and potential electron donors, with the sole addition of catalase at a concentration of 100 μg/mL. This setup comprised 4 μM *Tth*LPMO9G, 0.1% (w/v) PASC at pH 6, and 1 mM of the same electron donors being tested. Under these conditions, gallic acid emerged as the most effective electron donor, followed by caffeic acid, ascorbic acid, and then sinapic acid. It should be noted that the reactions under investigation lasted 16 h that constitutes sufficient time for electron donor-mediated H_2_O_2_ production, an important factor in this context. It is also worth noting that over the 16-h duration, an elevation in the amount of oxidized products from PASC oxidation was differentiated when catalase was included, as illustrated in Fig. 1 similar to finding shown before [31]. Catalase was added last to the reaction mixture and the experiment was conducted at a relatively low PASC concentration.

**Fig. 2:**
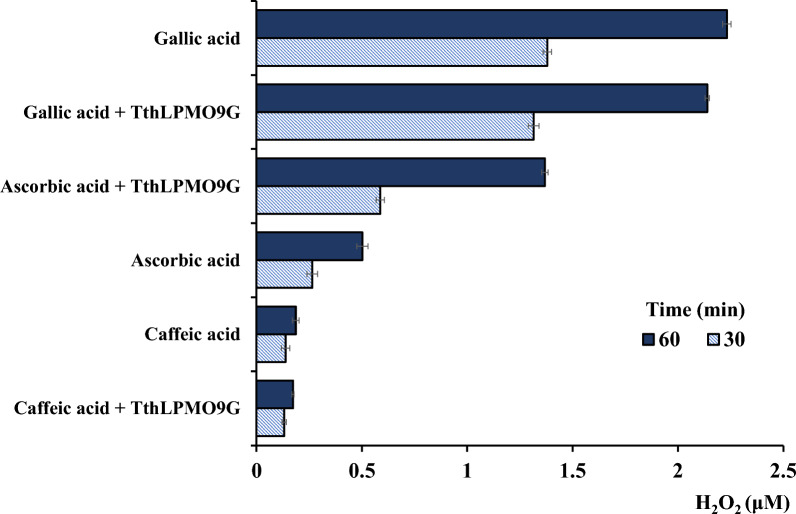
H_2_O_2_ accumulation in reactions with various electron donors in conjunction with *Tth*LPMO9G. The figure illustrates the concentrations of accumulated H_2_O_2_, apparent over a 1-h period from reactions with 30 µM of a freshly prepared electron donor and 0.65 µM LPMO. Concentrations were measured at 10 min intervals. In the bar chart, the light blue bars represent the H_2_O_2_ concentration measured at 30 min, while the dark blue bars correspond to the H_2_O_2_ concentration measured at 60 min. The labels specify if the enzyme was present in the reactions, and the samples are arranged in an ascending sequence. Reaction mixtures, prepared as per the guidelines of the Amplex® Red Hydrogen Peroxide/Peroxidase Assay Kit, included specific concentrations of buffer, temperature, HRP, and Amplex® Red. Error bars represent standard deviations among duplicate experiments, each conducted at least twice. Throughout the entire experimental setup, the concentrations of the standards—0.5 μM H_2_O_2_, 3 μM H_2_O_2_, 5 μM H_2_O_2_, and 10 μM H_2_O_2_—remained stable

After observing a shift in the optimal electron donor following the introduction of catalase, it was hypothesized that this alteration was somehow related to the depletion of H_2_O_2_. To validate this assumption, the H_2_O_2_ levels produced by each electron donor individually were subsequently assessed, as depicted in Fig. 2. In addition to the previously mentioned electron donors (caffeic acid, sinapic acid, ascorbic acid, gallic acid), other potential electron donors were also assessed for their capacity to produce H_2_O_2_ using the Amplex® Red protocol. Notably, gallic acid displayed a marked capacity to produce H_2_O_2_. This corroborates its correlation with H_2_O_2_ as observed in the earlier experimental setup involving catalase, where the depletion of H_2_O_2_ had the greater impact in the case of gallic acid. In terms of H_2_O_2_ release, it was followed by ascorbic acid.

LPMOs have oxidase activity and in the presence of electron donor, they may convert O_2_ to H_2_O_2_ [3, 22]. In analyzing the results from the 16-h reaction of *Tth*LPMO9G with PASC, H_2_O_2_ production was attributed only to electron donor’s source, and not to potential oxidase activity of LPMO, given the presence of the substrate. The substrate’s presence can insulate the copper from the solvent, displace water molecules, and alter the electronic environment of the metal ion. Yet, in the absence of a substrate, LPMOs might undergo uncoupled reactions post-reduction [16, 18]. One such uncoupled reaction could be the release of H_2_O_2_. Since our experimental setup was conducted over 16 h with 0.1% (w/v) PASC—a relatively low substrate concentration—it is plausible that the limited amount of substrate might allow for the occurrence of the LPMO oxidase reaction. Therefore, this potential side oxidase activity for *Tth*LPMO9G was also evaluated. Figure 2 also showcases *Tth*LPMOG9 in the presence of the electron donor, evaluating the release of H_2_O_2_. When LPMO is introduced, any additional H_2_O_2_ besides the electron donor-derived H_2_O_2_ observed can be attributed to its oxidase activity. This relationship was examined across all the electron donors. A notable result was observed when ascorbic acid was combined with LPMO; a 2.5-fold increase in H_2_O_2_ release compared to ascorbic acid alone was recorded. In contrast, no such pronounced H_2_O_2_ production was observed for any of the other electron donors. Previous studies made similar observations during their examination of LPMO oxidase activity in the presence of ascorbic and gallic acids. While they detected LPMO oxidase activity with ascorbic acid, none was observed with gallic acid. Surprisingly, despite the lack of detected oxidase activity, the LPMO catalytic rate on PASC was higher in the presence of gallic acid compared to ascorbic acid. This unexpected discrepancy was attributed to the inherent limitations of the HRP/Amplex® Red assay [29]. Figure 2 displays data specifically for gallic acid, with comparable observations noted for caffeic acid, sinapic acid, ferulic acid, and vanillin. To minimize potential interactions between HRP and the respective electron donor, electron donor concentrations of 30 μM were utilized, which were significantly lower than those of 1 mM employed in LPMO reactions, in accordance with reference [22]. The HRP displays a reductant peroxidase activity that consume H_2_O_2_, which thus cannot engage in the conversion of Amplex Red into resorufin. H_2_O_2_ levels are thus likely to be largely underestimated in the presence of residual reductant.

The insight into ascorbic acid’s dual capacity to produce H_2_O_2_ and induce LPMO’s oxidase activity prompted a re-evaluation of the initial findings from the 16-h experiment with low PASC concentration. As a result, the impact of catalase on ascorbic acid was specifically assessed during the initial half-hour time course. At this initial stage, one might hypothesize that the substrate concentration is sufficient to potentially preclude any oxidase activity by the LPMO. Additional file 1: Figure S1 demonstrates that when catalase was present, there was an increase in the oxidized PASC products within the first 30 min, similarly with the observations from the 16-h experiment.

Overall, the observed increase in oxidized PASC products upon the addition of catalase likely correlates with the prevention of H_2_O_2_-induced enzyme inactivation. In our experiments, we did not observe the inhibition of initial product formation under any of the tested reaction conditions, contrasting with the findings reported in literature [28, 30], probably due to the high concentration of catalase used in the reaction mixture. This provides a plausible explanation for the enhanced LPMO activity in the presence of the H_2_O_2_ scavenger. The study clearly demonstrates that among all redox partners analyzed, gallic acid results in the highest production of H_2_O_2_. This observation is validated by the data indicating a notable enhancement in product release from PASC oxidation when the H_2_O_2_ scavenger catalase is utilized, particularly in reactions involving gallic acid as compared to other electron donors. The observed increase in product release is probably due to decreased enzyme inactivation. This effect can be attributed to catalase, which removes H_2_O_2_—primarily generated by gallic acid—thus hindering the inactivation process.

**Fig. 3:**
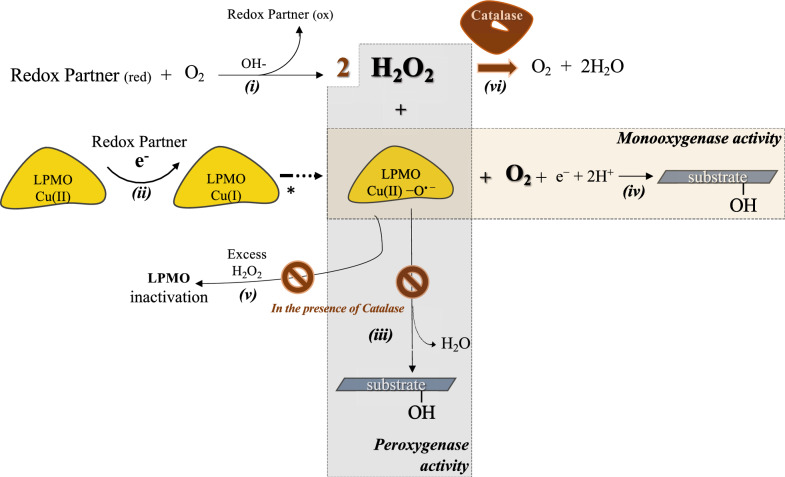
Representation of in vitro schematic potential catalytic LPMO pathways for polysaccharide substrate hydroxylation. Pathways are designated with Roman numerals (i–vii). *Pathway (i)*: reducing agents, substances that provide electrons and are subsequently oxidized, may produce non-enzymatically H_2_O_2_ over time, which may be intensified in certain conditions, such as high pH environment. *Pathway (ii)*: in this pathway, reducing agents reduce LPMO’s active site, transitioning the copper at the active site of the enzyme from its oxidized state of LPMO, Cu(II) is reduced to Cu(I) by redox partners, leading to the formation of the intermediate LPMO–Cu(II)-O_2_^–^. This species further transforms into LPMO–Cu(II)-OOH^–^, which, through interaction with another ascorbate molecule, resulting in the generation of H_2_O_2_ and Cu(I). Eventually, the Cu(I)- H_2_O_2_ intermediate converts to LPMO–Cu(II)-O^•^ ^–^, which is the final intermediate depicted in scheme (All the intermediates are indicated by an asterisk in the scheme). *Pathway (iii)*: this pathway demonstrates the peroxygenase H_2_O_2_-driven activity of LPMOs for the hydroxylation of polysaccharide substrates (e.g., cellulose). *Pathway (iv)*: τhe monooxygenase activity of LPMOs is driven by molecular O_2_. *Pathway (v)*: LPMO-catalyzed reactions can undergo enzyme inactivation, likely due to auto-oxidation, a process where the LPMO becomes oxidized and thus loses its activity. Reduced LPMOs, whether the reaction is driven by O_2_ or H_2_O_2_, are vulnerable to oxidative self-inactivation, especially in the presence of excess H_2_O_2_ concentrations. *Pathway (vi)*: catalase effectively decomposes H_2_O_2_ into water and O2, significantly mitigating the risk of oxidative damage. This action minimizes or eliminates H_2_O_2_—dependent pathways, such as *Pathway (iii)*, altering the possible reactions that LPMOs can partake in. Catalase interfered reactions are presented with brown colored shapes

All feasible pathways, encompassing every component present in the LPMO-mediated system and the combined catalase-LPMO system, are delineated in Fig. 3. In this in vitro scheme, LPMO is presented in the presence of a substrate, so it does not depict the oxidase activity of LPMOs that results in H_2_O_2_ production. The oxidase reaction typically occurs when the substrate is absent from the reaction mixture.

As reported in literature, the addition of catalase instigates a delay in enzyme inactivation [16]. When an LPMO is in its reduced state and encounters H_2_O_2_ without a substrate, or if substrate concentrations are deemed insufficient, or when the binding to the substrate is assessed as weak or imprecise, an inefficient use of H_2_O_2_ might be detected. This can lead to the generation of H_2_O_2_, potential oxidation of the active site, and possible subsequent inactivation of the enzyme [16, 34]. In both monooxygenase and peroxygenase pathways, the enzyme undergoes deactivation. Notably, higher concentrations of H_2_O_2_ intensify this deactivation phenomenon. In conjunction with findings reported by Goltén et al. [28] showing that higher concentrations of catalase lead to increased maximum product yields, implies that the regulation of H_2_O_2_ concentration by catalase can enhance the efficiency of LPMOs. This observation aligns with Scott et al. findings which noted a beneficial effect of catalase addition on the enzymatic saccharification of lignocellulosic biomass with LPMO-containing cellulase cocktails [35]. This differential capacity may influence the effect of catalase on LPMO reactions, leading to a dual effect. On one hand, catalase reduces the reaction rate by limiting the availability of H_2_O_2_, which is critical for LPMOs. On the other hand, catalase potentially enhances the overall efficiency and stability of the LPMO reaction by regulating H_2_O_2_ concentration and thus mitigating destructive off-pathway reactions. The interplay of these elements paints a complex scenario where multiple possible LPMO pathways can be selectively limited by controlling the combination of LPMO and catalase, primarily focusing on monooxygenase activity.

### Rational design and mining of *Tth*LPMO9G mutations

The subsequent section focuses on the rational design targeting amino acids that coordinate the active site. Notably, the LPMO active site is adeptly structured for controlled metal-catalyzed generation of highly oxidative oxygen species. Recent studies have expanded focus from amino acids directly interacting with copper and oxyl radicals (first coordination sphere) to also include those in the surrounding area (secondary coordination sphere). For instance, a recent study posited that the elevated experimental reoxidation rate observed for the glutamate residue found in the second coordination sphere could be rationalized if this residue is protonated [12]. The proposition that oxyl radicals interact more with the amino acids of the secondary coordination rather than with copper or primary coordination amino acids is intriguing as well [36]. A point of particular argument and interest is the secondary coordination histidine which interacts with His1 of the active site as illustrated in Table 1, insights gained around this second coordination histidine encompass aspects like cellulose recognition [15, 37, 38], glycosidic bond destabilization through a potential catalytic elimination reaction oriented by a conserved third histidine [37], H_2_O_2_ stabilization [36], and possible involvement in proton transfer [39–41]. H_2_O_2_ is stabilized and coordinated by the amino acids of the second sphere. The amino acids closest to H_2_O_2_ are second-sphere glutamine and second-sphere histidine, with histidine being 2 Å away from H_2_O_2_ [36]. Consequently, this region was spotlighted for our study, leading to a histidine to alanine substitution. Previous studies have examined this specific substitution demonstrating a decrease in cellulose oxidation [15]. It is important to note that the mutation study mentioned above was conducted before the discovery of LPMO’s capability to function as peroxygenases. Consequently, it did not explore the effects of oxidase and peroxidase activities.

**Table 1:** Analysis of the histidine in the second coordination sphere of AA9 LPMOs

PDB name	Microorganism	% Identity with* Tth*LPMO9G	Position of histidine in Sequence	Discussed role	References
*Tth*LPMO9G	*Thermothelomyces thermophilus*	100.0	H140	Mutagenesis of H140A that indicates: ↓monooxygenase/peroxygenase activity ↓peroxidase like activity ↓oxidase activity	Current & [30]
4B5Q	*Phanerodontia chrysosporium*	41.4	H149	MD simulation for cellulose recognition	[38]
4D7U	*Neurospora crassa*	36.8	H155	Direct interaction of a cellobiose dehydrogenase with the LPMO	[42]
4EIR	*Neurospora crassa*	36.9	H157	Destabilization of the glycosidic bond with the elimination reaction possibly catalyzed by a conserved third histidine Also identify the conserved second coordination shell residue His157 as the proton donor	[37, 41]
4EIS	*Neurospora crassa*	37.1	H160	Destabilization of the glycosidic bond with the elimination reaction possibly catalyzed by a conserved third histidine	[37]
4UFV	*Thermothelomyces thermophilus*	37.0	H161	Mutagenesis of H161A that indicates reduction in cellulose oxidation activity. Stabilizing bound oxygen and possible participation in proton transfer	[15]
5ACF	*Panus similis*	34.0	H147	QM/MM metadynamics simulations with doubly protonated His147 show that such reaction does not lead to a stable product, ruling out H_2_O_2_ formation via the proton transfer pathway Also is generally singly protonated thus cannot be a proton donor	[39, 40]
5NNS	*Heterobasidion irregulare*	34.5	H159	The active site was defined as His1, Pro79, His80, His159, Gln165, Tyr167, one crystallographic water molecule and the copper ion	[43]
5O2X	*Hypocrea jecorina*	32.6	H-163	H_2_O_2_ connects to amino acids in the second sphere. The amino acids closest to H_2_O_2_ are second-sphere glutamine and second-sphere histidine, with histidine being 2 Armstrong away from H_2_O_2_	[36]

The table summarizes various AA9 LPMOs, highlighting the exact position of the second coordination sphere histidine. For each LPMO, the table lists its Protein Data Bank (PDB) identifier, the source microorganism, it is percent identity with *Tth*LPMO9G, the position of histidine in its amino acid sequence, the discussed role of the histidine, and the corresponding reference. This compilation allows for a comparative analysis of histidine’s role in different LPMOs, providing insights into the similarities and variations in their functional mechanisms

In the case of *Tth*LPMO9G, the second coordination histidine is located at amino acid position 140, representing a highly conserved residue within AA9 LPMOs, underscoring its vital significance in enzyme function or structure across various species [30]. Positioned right after the 8th barrel in a bend of the protein structure, His140 lies of the *Tth*LPMO9G active site. It is 5.6 Å from the coordinated copper and 3.3 Å from His70, the amino acid that participates in the formation of the characteristic ‘histidine brace’ (Fig. 5).

**Fig. 4:**
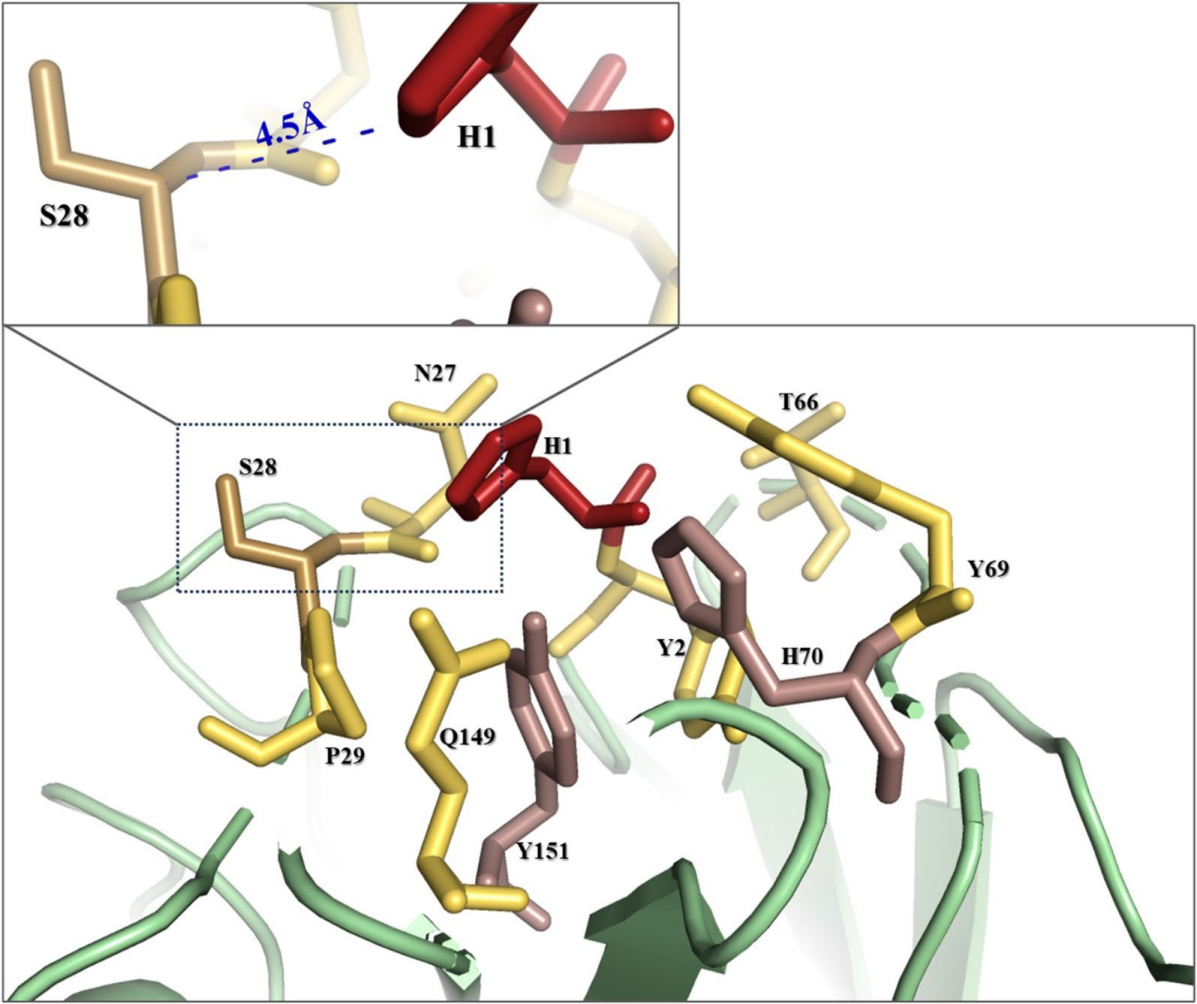
Representation of the AlphaFold model for *Tth*LPMO9G rational design mining of selecting Ser28 as a mutation substitution location. The active site is prominently showcased, with focus on the location where Ser28 is situated relative to the adjacent histidine His1. Active site amino acids are colored in red, while residues within a 5 Å radius around His1 are in yellow. The dashed blue line represents the distance measured using the AlphaFold wizard. Distances are derived from the nearest atoms between these specific residues

The deeper we understand the active site structure and its interactions, the clearer it becomes that crucial amino acids are involved in the histidine brace interactions. Narrowing down further our focus, we can pinpoint histidine 1 as perhaps the most pivotal player in this context. Numerous studies have highlighted that apart from coordinating the copper, His1 undergoes methylation [44] a feature that seems to shield it from autocatalytic inactivation. Additionally, His1, being solvent-exposed, establishes very close interactions with the cellulose substrate [45]. Wang et al.’s molecular dynamics simulations indicated that, in the presence of an electron donor (specifically ascorbic acid) and substrate, His1 was the residue most proximal to the electron donor interaction [39]. Although the significance of His1 is unquestionable, direct substitution attempts in the past led to the abrogation of LPMO activity as demonstrated by Harris et al. [14]. In our rationale approach to probe the vicinity of His1 depicted in Fig. 4, exploration based on the AlphaFold model of *Tth*LPMO9G, seven amino acids were discerned within a 5 Å radius of His1. Evaluating these candidates for potential mutation presented a series of considerations. Firstly, four of these amino acids were immediate sequential neighbors of the active sites’ residues in the protein’s primary sequence. Disturbing these could potentially introduce unintended perturbations in the protein’s biochemistry, so they were deemed unsuitable targets. On the other hand, the remaining three residues formed part of the Loop 2 motif, characterized by the sequence “NSP”. Intriguingly, both the asparagine and proline showcased high conservation, suggesting their possible roles in maintaining protein stability and function [30]. Thus, to avoid undermining the stability and structural features of the protein, we opted not to alter these conserved residues. This line of reasoning left the serine, situated between N and P, as the most viable candidate. Targeting Ser28 for mutation could offer a nuanced alteration in the interaction dynamics between Ser28 and His1, allowing us to estimate its effect without disruption to the protein’s core architecture. In the structural analysis of the enzyme, the serine and histidine residues exhibit a critical interaction pertinent to the enzyme’s function. The distance between the carbon atoms of the respective residues is measured at 4.5 Å, suggesting a proximity that may be indicative of a catalytic or structural role. More specifically the distance between the beta carbon (Cβ) of Ser28 and the presumed delta carbon (Cδ) or epsilon carbon (Cε) of His1 (H1) is 4.5 Å. While the exact residue may not be conserved, the spatial configuration of loop 2 and the implications for enzyme function we observed are pertinent to the AA9 family.

### Effect of H140A and S28A *Tth*LPMO9G substitutions on oxidase and peroxidase activities

In this study, we sought to evaluate the enzymatic profiles of both the H140A and S28A mutants, particularly their peroxidase, oxidase, and LPMO monooxygenase/peroxygenase activities.

**Fig. 5:**
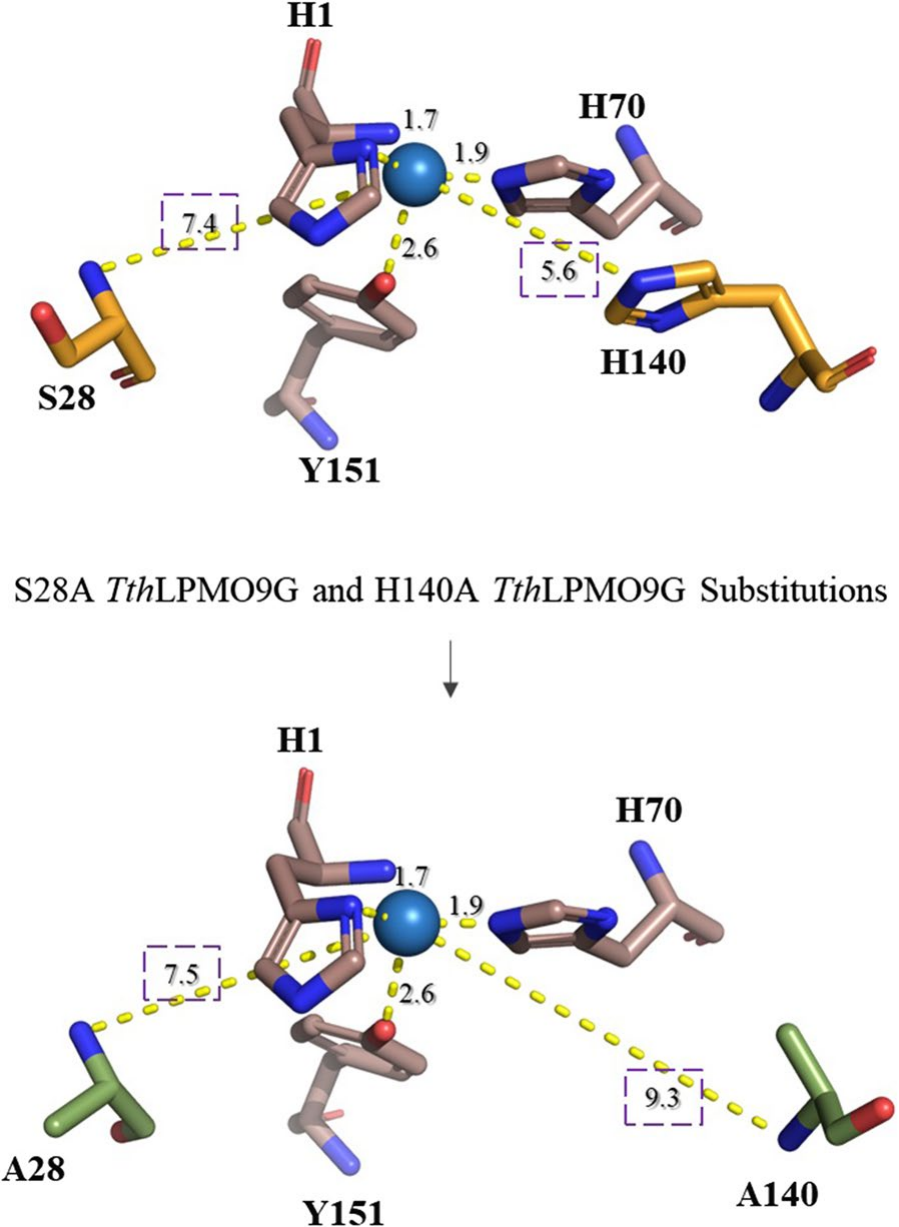
AlphaFold model representation of *Tth*LPMO9G, specifically showcasing the active site and portions of the second coordination sphere where Ser28 and His140 are located. The active site and relevant surrounding residues were visualized and analyzed using the PyMOL molecular visualization system. The lower image shows the modeled single-point mutations S28A and H140A. Single-point mutations were introduced in silico using the mutagenesis wizard in PyMOL, which allows for the substitution of selected amino acids. Distances between the nitrogen atoms of the mutated residues and the copper ion in the active site were measured within PyMOL to assess changes in the coordination sphere resulting from the mutations

In an earlier study of *Tth*LPMO9G [30] multiple alignment revealed that the *Phanerochaete chrysosporium* GH61D enzyme (PDB: 4B5Q; [38] was the most homologous structure to *Tth*LPMO9G within the PDB database. Interestingly, the publication detailing the structure of GH61D emphasized the significance of a second coordination sphere histidine. Molecular dynamics simulations from this study pinpointed this histidine as a key residue in cellulose interaction. *Tth*LPMO9G was superimposed on the GH61D structure using PyMOL. The root mean square deviation (RMSD) value was determined to be 0.654 Å, calculated based on the alpha carbons (CA atoms) of the two protein structures being compared. This analysis specifically focused on the 201 amino acids of *Tth*LPMO9G and 200 amino acids of 4B5Q beginning with His1, while deliberately excluding the signal peptide, the C-terminal region, and the carbohydrate-binding module (CBM) 1. This specific region was chosen for comparison because this is the area where AlphaFold predicted the protein structure with high confidence, as depicted in Chorozian et al. [30] underscoring the structural similarity between the two. The superimposition is graphically depicted in Additional file 1: Figure S2, validating the accuracy of our predicted model through this structural comparison. Figure 5 illustrates the altered spatial relationships in the *Tth*LPMO9G H140A and *Tth*LPMO9G S28A variants, with an emphasis on the distances introduced by these mutations in proximity to the copper of the active site.

The *Tth*LPMO9G variants were expressed using the molecular engineering protocols detailed in the materials and methods section, with the set of primers showcased materials and methods. The yields of purified *Tth*LPMO9G and its variants reached 42.3 mg for the *Tth*LPMO9G, 16.8 mg for the S28A and 52.9 mg for the H140A variant per liter of culture supernatant. SDS-PAGE analysis confirmed the enzymes’ homogeneity, represented by single band of 55 kDa (Additional file 1: Figure S3).

The oxidase and peroxidase activities of *Tth*LPMO9G and its mutants were studied using the Amplex® Red and the 2,6-dimethoxyphenol (2,6-DMP) assay, respectively, as shown in Table 2. These findings emphasize the adverse impact of the H140A mutation on enzymatic activities, with decrease of approximately 30% and 25% of the WT strain observed in peroxidase and oxidase activities, respectively. This outcome was expected and can be linked to the decreased ability of the enzyme to recognize H_2_O_2_ as a co-substrate and diminished H_2_O_2_ release activity as two separate side activities. Notably, a study published a year ago highlighted this association, revealing a mere 2 Å distance between H_2_O_2_ and the second-sphere histidine [36]. As well as this histidine’s role to act as proton donor for the formation of H_2_O_2_ might influence the *Tth*LPMO9G ability to use H_2_O_2_ (peroxidase activity) or O_2_ (oxidase activity) as co-substrate [41]. However, a study released in the same period challenged the notion that second-sphere histidine can act as a proton donor [40].

**Table 2:** Oxidase and peroxidase activity assays for *Tth*LPMO9G and its point mutations

Assay	Activity			Notes
	WT	S28A	H140A	
Amplex® Red^a^	1.67 ± 0.2 (U/mg)	1.37 ± 0.1 (U/mg)	1.01 ± 0.1 (U/mg)	A decrease of 18% and 40% was observed in activity, respectively, compared to the WT
2,6-DMP^b^	11.5 ± 0.5 (U/g)	10.6 ± 0.4 (U/g)	8.3 ± 0.2 (U/g)	A decrease of 8% and 28% was observed in activity, respectively, compared to the WT

^a^Oxidase activity, indicating H_2_O_2_ production, is measured in μM/min/g of enzyme using the Amplex Red® assay

^b^Peroxidase activity, reflecting coerulignone generation, is quantified in units/min/mg of enzyme using the 2,6-DMP assay

Slight changes in the interaction with H_2_O_2_ were observed with the S28A mutation, also presented in Table 2. Comparing the H_2_O_2_ release rate from WT *Tth*LPMO9G with previous studies on LPMOs presents challenges due to the variability in experimental setups between different studies. Variations in experimental conditions across different studies hinder direct comparisons, necessitating cautious interpretation of data. Further matters related to this issue are discussed in a recently published review paper [46].

The peroxidase activity of WT *Tth*LPMO9G has been previously discussed and compared with other LPMOs’ peroxidase activities [30]. The present study exclusively focuses on comparing the peroxidase activity via 2.6-DMP assay between the mutant variants. Additional file 1: Figure S4B presents real-time values gathered from our experimental setup, with details outlined in the Materials and methods section.

### Effect of H140A and S28A substitutions on LPMO monooxygenase/peroxygenase activity on cellulose

**Fig. 6:**
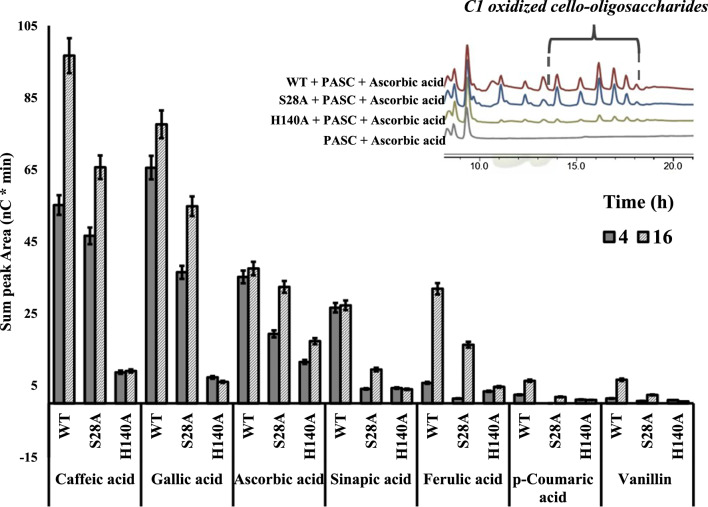
Comparative analysis of total product release (in nC*min) resulting from cellulose oxidation by *Tth*LPMO9G variants, following 4 h (dark grey) and 16 h (light striped bars) reaction periods using various electron donors. The experiment comprises the WT *Tth*LPMO9G and two mutations, S28A and H140A. Each reaction incorporated a 0.1% w/v PASC concentration, 4 μΜ LPMO and 1 mM electron donors. All experiments were carried out in 50 mM sodium acetate buffer, pH 6.0, at 45 °C. The bar chart illustrates the comparative data of eluted products for WT and the mutants with each respective electron donor. The measured areas correspond to the peaks between 13–19 min, which is the elution window for C1 oxidized products. The bars represent mean values, with error bars indicating the standard error derived from two independent experiments, each conducted at least twice. All control reactions without enzyme addition resulted in zero area

The peroxygenase and monooxygenase activity of WT *Tth*LPMO9G and its mutant variants, S28A and H140A, were examined on a 0.1% (w/v) PASC cellulosic substrate. This analysis was modeled on the experimental setup outlined in Fig. 1 but expanded to include reactions at two distinct time intervals, namely 4 and 16 h, using 10 different electron donors. Figure 6 in the present study illustrates the comprehensive setup and results of the reactions with various donors in these two distinct time intervals, facilitating a direct comparison between the activities of WT *Tth*LPMO9G, S28A, and H140A. The electron donors shown in Fig. 6 represent only those which demonstrated potential as electron donors and exhibited product release, at least in the WT. The structures and chemical classification of the organic compounds that successfully served as electron donors for *Tth*LPMO9G are detailed in Additional file 1: Table S1.

Figure 6A illustrates the capacity of 7 out of 10 tested electron donors to generate increased amounts of released oxidized products, with caffeic acid demonstrating the greatest capacity, followed by gallic acid, and then ascorbic acid. On the other hand, no products were released in the presence of 2-hydroxybenzoic acid, methyl-3,4,5-trihydroxybenzoate, or vanillic acid, so these are not depicted in the chart.

For the WT the 16-h pattern revealed the sequence of redox partners from most effective to least as; caffeic acid > gallic acid > ascorbic acid > ferulic acid > sinapic acid > vanillin > *p*-coumaric acid. In the 4 h time course, the sequence was: gallic acid > caffeic acid > ascorbic acid > ferulic acid > sinapic acid > vanillin > *p*-coumaric acid. Interestingly, the S28A pattern for redox partners, from most to least effective, remains consistent. In contrast, H140A shows a time-increasing product pattern exclusively with ascorbic acid as the electron donors. For all other redox partners, H140A’s activity appears nearly depleted, with no discernible increase in rate.

When comparing the results at 4 and 16 h for the WT with most electron donors, there is an observed increase in product formation. However, this trend is not apparent with ascorbic acid and sinapic acid, suggesting a different pattern at these two time intervals. This distinct pattern may be due to potential autocatalytic inactivation of the enzyme in these specific cases. It is also worth noting the reaction with ferulic acid and WT, which shows a significant 4.5-fold increase in product between the 4- and 16-h marks. In contrast, caffeic acid showed a 1.75-fold increase, while ascorbic indicated a decrease of 5%. This variability in reaction behavior or product formation warrants further investigation. One potential explanation could be enzyme inactivation.

When comparing the products across all donors and mutant variations, S28A showed a marked decrease in the product pattern, which was even more evident for H140A. Notably, S28A had a reduced product release from PASC compared to the WT. After a 16-h reaction, there was a notable drop in product release for both caffeic and gallic acid in S28A relative to the WT. Ascorbic acid also displayed a decrease, while the reduction in other electron donors for S28A was substantially greater compared to WT. Next, a detailed analysis of product patterns for S28A was conducted, with a focus on the electron donors; ascorbic acid, gallic acid, and caffeic acid. This was not extended to sinapic acid, ferulic, *p*-coumaric acid, and vanillin because their product reduction, when compared to the WT, exceeded 50% and the products released, lacked statistical significance in quantification.

**Fig. 7:**
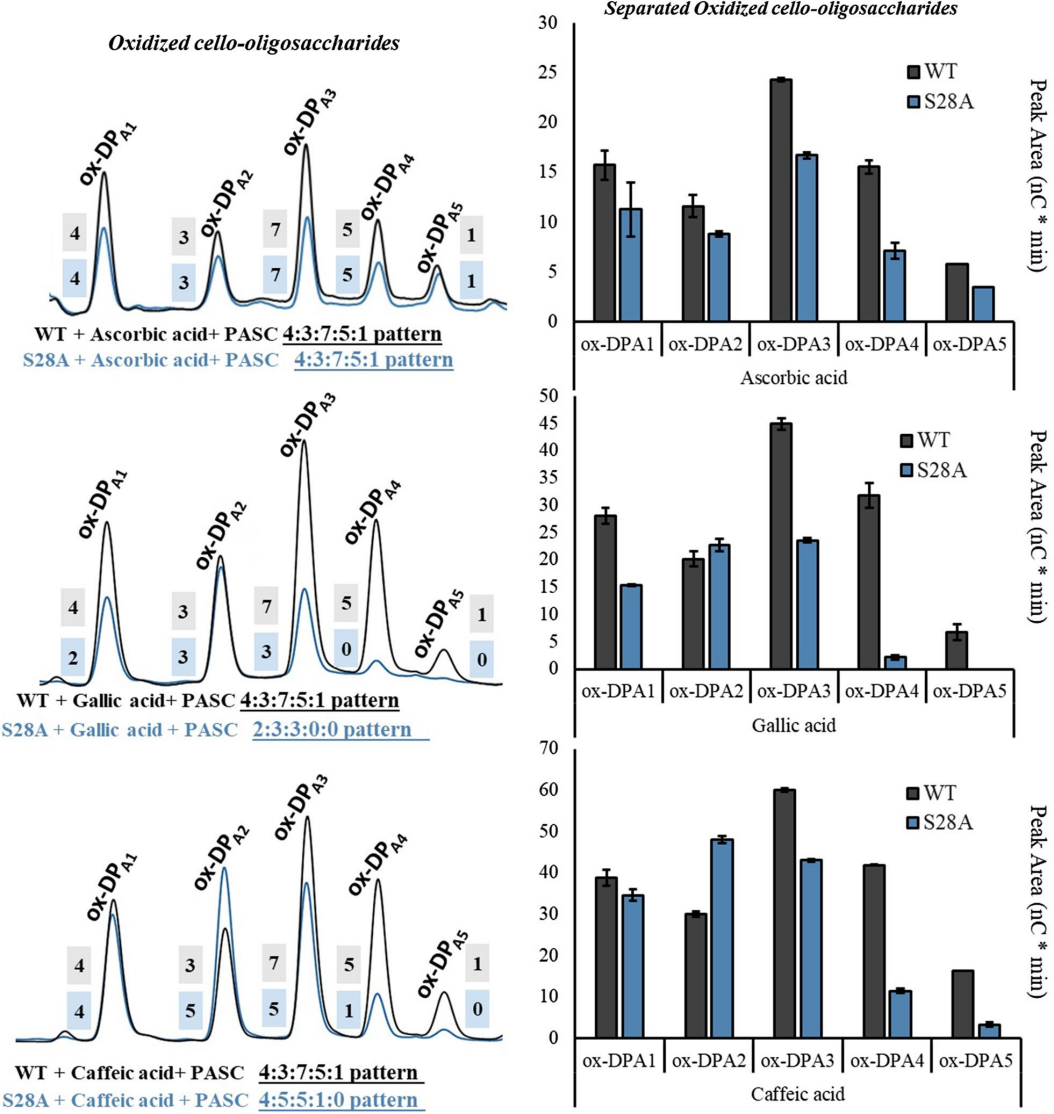
Comparative analysis of individual product release (in nC*min) from cellulose oxidation catalyzed by TthLPMO9G variants, WT (dark grey) and the S28A mutant (blue), following a 16-h reaction period. Each reaction incorporated a 0.1% PASC concentration, 4 μΜ LPMO, and 1 mM electron donors, and was diluted threefold with water prior to analysis via ion chromatography HPAEC-PAD. All experiments were carried out in 50 mM sodium acetate buffer, pH 6.0, at 45 °C. The chromatograms on the left delineate all eluted products, labeled ox-DP_A1_ to ox-DP_A5_ for the 13–19 min retention window. The bar chart on the right presents a comparative analysis of each eluted product for the WT and the S28A mutant under the influence of ascorbic acid and both gallic and caffeic acids. Control reactions without the enzyme addition consistently resulted in zero area. Product patterns ratios were derived from summed peak areas and normalized to the smallest value. Bars denote mean values, with error bars indicating the standard error derived from two independent experiments, each performed at least twice

In-depth analysis of the products released, demonstrates varying outcomes contingent on the electron donor used. Starting with, reactions incubated for 16 h were analyzed through HPAEC-PAD and subsequently quantified in the region of C1 oxidized products. Five characteristic products with elution times of 14, 15.5, 16.2, 17 and 18 min are referred to this study as ox-DP_A1_ to ox-DP_A5_. In the reactions where WT and S28A acted on PASC, using ascorbic acid as the electron donor, a decrease in the overall quantity of products was observed for the S28A mutant. A more detailed examination of this decrease is provided through the individual quantification of each product, illustrated in Fig. 7. The pattern of C1 products from ox-DP_A1_ to ox-DP_A5_ in the WT and S28A for ascorbic acid as electron donors displayed a product area ratio of 4:3:7:5:1 (corresponding to ox-DP_A1,_ ox-DP_A2,_ ox-DP_A3,_ ox-DP_A4_ and ox-DP_A5_, respectively) after product patterns ratios were derived from summed peak areas and normalized to the smallest value. The ratios of oxidized products from ox-DP_A1_ to ox-DP_A5_ exhibited significant variations between the WT and the S28A mutant when gallic and caffeic acid were used as redox partner in reactions with PASC as a substrate. As depicted in Fig. 7 the product pattern ratio of the S28A mutant shifted to 2:3:3:0:0 in the case of gallic acid, and to 4:5:5:1:0 when caffeic acid was used. It should be noted that that the common pattern for the soluble products ratio, namely the 4:3:7:5:1, was observed for S28A only when ascorbic acid was used as donor.

The above reaction product pattern analysis was investigated in time course reactions of 1, 4 and 16 h. In Fig. 7, the chromatograms from the 16-h reactions are presented, while Additional file 1: Figure S5 depicts the products from 1 and 4 h reactions for gallic acid only. Similar patterns were observed for caffeic acid. The primary observation is that the peak areas of the specific product pattern appear to increase linearly over time.

The S28A substitution has the potential to modify interactions within the second coordination sphere. This sphere is crucial for the electron transfer from the electron donors to the active site’s copper, a process which transforms the copper state from Cu(II) to Cu(I) and might influence substrate recognition and oxidation catalysis [39].

In a recent molecular dynamics study Wang et al. examined LPMOs from *Lentinus similis*, specifically *Ls*LPMO9Α [39]. Their results indicated that the electron transfer from ascorbic acid to the active site occurs in close proximity to His-1 of the active site. That study using QM/MM MD simulations has highlighted that ascorbate with its redox-active hydroxyl group tends to form a hydrogen bond with the proximal oxygen of Cu–OOH. Moreover, the ascorbate establishes bonds with the oligosaccharide substrate, which is a difference among the reference study and our study which is important to mention. However, such interactions offer insights into the electron transfer process among the active site, substrate and electron donors. Regarding the *Tth*LPMO9G of the current study, Ser28 is located approximately 4.5 Å away from the first histidine as well, maintaining a similar orientation as observed in the *Ls*LPMO9Α. Drawing from the principles of the inner-sphere electron transfer mechanism, where a direct coordination bond or bridging ligand exists between the electron donor and acceptor, we hypothesize that the mutation substitution S28A might disrupt the electron donor’s interaction. Given this mechanism, our targeted mutation, S28A, might influence a region that concurrently affects the interaction among the LPMO active site, the electron donors, and the substrate. This structural correlation becomes more evident when considering the distinct product patterns of *Tth*LPMO9G S28A displayed by different electron donors. Specifically, dihydroxybenzene (caffeic acid) exhibits a pattern distinct from trihydroxybenzenes (e.g., gallic acid), which also differs from γ-lactones such as ascorbic acid. Given these findings and the questions posed, tailored molecular dynamics simulations for our system could offer valuable insights into the electron transfer dynamics. We recommend further exploration through such simulations in future research endeavors since this approach could be instrumental in understanding its oxidative activity, especially when evaluating the effects of structural alterations in the enzyme. Our primary hypothesis suggests that if electron donors bond with both the active site and the substrate, then specific amino acids, like Ser28 in this instance—or His1 which has been already known—could play a crucial role in these interactions.

Furthermore, redox partners play multiple roles in the total LPMO catalytic cycle. Specifically, electron donors such as ascorbic acid, not only serve as electron donors for the initial copper reduction, but also emerge as the most probable candidates for both proton and hydrogen donation. There are simulations searching the catalytic cycle, and examine the LPMO–Cu(II)-O_2_^–^ abstraction of hydrogen atom (HAA) from ascorbate. Subsequently, Cu(II)–OOH^–^ extracts a hydrogen atom from another ascorbate molecule, resulting in the formation of H_2_O_2_ and reverting to Cu(I) [39]. Hence, in one full cycle, ascorbic acid undertakes three distinct roles: one for electron donation and two as HAA. This implies redox partners might influence the process not only as electron donors but also as proton donors. This makes the initial results more challenging to interpret due to the presence of multiple contributing aspects.

Regarding S28A point mutation results, previous studies on mutations within the Loop 2 region have posited its role in determining regioselectivity [47, 48]. A discovery was made in a study of an LPMO mutant from *Neurospora crassa* where it was observed that a mutant which lacked crucial components of the Loop 2 region, largely forfeited its ability to oxidize at the C4 position [47]. Similarly, Danneels et al. reported that substitutions of two surface-exposed aromatic residues near the L2 region of *Hj*LPMO9A led to variants exhibiting increased production of C1-oxidized cellulose products [49]. In contrast, a variant with an alanine substitution for loop 2 amino acid, produced more C4-oxidized products. Our hypothesis, grounded on previous simulation findings, posited that the S28A region, located near His1, would interfere with the active site–substrate–electron donor interaction. Indeed, our results suggest a modified mode of action in this region, highlighting its crucial role in determining product specificity conjugated electron donor specificity. Electron donors like gallic acid present the S28A *Tth*LPMO9G variant demonstrated increased substrate specificity, as evidenced by the release of more specific products of ox-DP_A1_ to ox-DP_A3_, as depicted in Additional file 1: Figure S5 as well. This contrasts with the release of less specific products observed with the S28A *Tth*LPMO9G variant in the presence of ascorbic acid of ox-DP_A1_ to ox-DP_A5_.

Finally, the study focused on assessing the enzyme variants’ ability to bind to cellulose. The binding assays commenced with the addition of either WT *Tth*LPMO9G or its variants to a final concentration of 10 μΜ with 1 mg/mL PASC. After 1-h incubation and subsequent centrifugation, the substrate was effectively separated from the protein content in the solution. Control samples, which included only the enzyme and buffer, established a 100% unbound protein, while an additional control consisting solely of PASC without protein was used to calibrate the absorbance readings at a wavelength of 280 nm (A280). Analysis of the A280 absorbance data revealed a 44.6 ± 1% decrease in the free solution protein content for the WT, which was attributed to the protein binding to PASC, since *Tth*LPMO9G possess a CBM of family 1 [30]. The H140A and S28A mutants exhibited decreases in free solution protein content of 33.6 ± 1.5% and 38.8 ± 1.2%, respectively. Relative to the wild type, the decrease in binding capacity for H140A was calculated to be 11 ± 0.3%, and for S28A, it was 5.8 ± 0.2%. These percentages are indicative of a minimal influence of the mutations on the enzyme’s substrate binding efficiency. This is consistent with another study where mutations assessed for substrate binding also showed comparable declines [50].

## Conclusions

This research paper presented a detailed comparative study of the effect of different electron donors on the activity of LPMOs on cellulose, with a particular focus on identifying the most efficient donor under commonly studied conditions employed in LPMO research studies throughout the literature. We introduce catalase to exclude the interference from excess H_2_O_2_ produced by electron donors, thereby ensuring that the observed efficacy of electron donors is solely based on their ability to deliver electrons to the LPMO active site without the confounding influence of H_2_O_2_. This approach allows for a more accurate evaluation of electron donor efficiency in facilitating LPMO reactions with cellulose. In the *Tth*LPMO9G reaction on PASC as a substrate, caffeic acid was identified as the most effective electron donor. However, this dominance shifted in favor of gallic acid when catalase was introduced. Notably, catalase generally augmented the release of oxidized PASC across all electron donors tested. The strategic use of catalase to mitigate H_2_O_2_ levels unveiled its dual potential impact, since H_2_O_2_ can either act as an enhancing co-substrate for peroxygenase activity or, at elevated concentrations, lead to enzyme inactivation. The enhanced cellulose products release observed post H_2_O_2_ by catalase depletion leans towards the latter hypothesis, suggesting prevalent enzyme inactivation. Among the electron donors, gallic acid stood out for its high H_2_O_2_ production capabilities, while only ascorbic acid-initiated oxidase activity on *Tth*LPMO9G. Using rational design methodologies, we delved into the active site’s amino acid environment, fostering profound insights into the aforementioned interactions. The H140A *Tth*LPMO9G mutant displayed a significant decrease in both oxidase and peroxidase activities, with a substantial drop in LPMO monooxygenase/peroxygenase activity on PASC. However, it displayed markedly increased amounts of glucose product release. On the other hand, S28A *Tth*LPMO9G demonstrated a smaller decrease in these activities, the magnitude of which was electron donors dependent. When compared with the WT, S28A *Tth*LPMO9G revealed a distinct product pattern profile for gallic and caffeic acid, but retained a consistent (even though weakened) pattern for ascorbic acid. The insights from the S28A substitution provided valuable contributions, especially when analyzed through theoretical perspectives predicting substrate and electron donor interactions, possibly due to the His1-Ser28 environment. This has contributed to the identification of a new association between the enzyme, electron donor, and product pattern, marking an initial observation in this area of research.

## Methods

### Effect of various electron donors on LPMO-mediated cellulose oxidation

Cleavage assays were carried out in 500-μL aliquots of buffered solution of 50 mM sodium acetate–acetic acid pH 6, containing 0.1% (w/v) PASC in apparent monooxygenase reaction conditions. In the reactions with LPMO, the sum of integrated peak areas of C1-oxidized products (nC * min) was used as a for LPMO activity as previously described [51]. PASC was prepared from Avicel (Sigma-Aldrich) according to the method described previously [52]. Reaction mixtures consisted of *Tth*LPMO9G 4 μM, and concentration 1 mM of freshly prepared electron donors including caffeic acid, gallic acid, ascorbic acid, sinapic acid, ferulic acid, *p*-coumaric acid, vanillin, methyl- 3,4,5-trihydroxybenzoate, vanillic acid and 2-hydroxybenzoic acid. The above organic compounds were purchased by Sigma-Aldrich and prepared by dissolving them in water, using the minimal amount of DMSO necessary to achieve solubilization. Samples were incubated for 1, 4 and 16 h in an Eppendorf Thermomixer comfort at 45 °C and 900 rpm. In the same reaction conditions, catalase from bovine liver was incorporated, obtained from Sigma, and utilized at a concentration of 100 μg/mL. The product is characterized by a potency of 2440 units/mg solid. The reactions were stopped after a 5-min incubation at 100 °C. The insoluble fraction was separated from the soluble products by centrifugation of the samples at 15,000×*g* for 10 min and the soluble products were analyzed by HPAEC-PAD, as described below. The aforementioned activity of LPMO acting on cellulosic substrates is referred to as monooxygenase/peroxygenase activity.

### LPMO activity assays

The production of H_2_O_2_ by the LPMO was measured using the Amplex® Red Hydrogen Peroxide/Peroxidase Assay Kit purchased by Invitrogen Thermo Fisher Scientific. The reaction mixtures (100 μL) consisted of 50–12.5 nM LPMO, 30 μM electron donor, 0.5 U horseradish peroxidase (HRP) and 100 μM Amplex® Red in 50 mM sodium phosphate, pH 7.4. Excitation was set to 530 nm, and fluorescence emission detection was conducted at 590 nm. The reaction was monitored over time by a Tecan Infinite M1000 Pro fluorescence microplate reader at 30 °C for 60 min, with 10 min interval time, while analysis was conducted with Tecan i-control software. The aforementioned activity of LPMO producing H_2_O_2_ is described as oxidase activity.

To assess the peroxidase activity of the LPMOs, an assay was used that as described previously [53]. The assay consisted of a reaction mixture containing 1–4 μM of enzyme, 10 mM freshly made 2,6-DMP (Sigma–Aldrich, St. Louis, MO, US), 500 µM H_2_O_2_ and 50 mM sodium acetate buffer pH 6.0. Control reactions were prepared either in the absence of enzyme or by using denatured enzyme previously incubated at 99 °C for 10 min before added into the reaction mixture. 2,6-DMP was pre-incubated with buffer at 40 °C for 15 min prior to LPMO addition and the formation of reaction product, coerulignone, was monitored kinetically for 10 min as an increase in absorbance at 469 nm. The linear part of the curve (*R*^2^ > 0.998) was used to calculate the *V*_max_ as an estimation of LPMO’s ability to oxidize the electron donors. The aforementioned activity where LPMO utilizes H_2_O_2_ as a co-substrate is identified as peroxidase activity.

### Site-directed mutagenesis, protein production and purification

In the context of this study, we clarify the nomenclature used for the enzyme under investigation to avoid confusion with previously studied enzymes. Another LPMO named *Tt*LPMO9G, derived from *Thermothielavioides terrestris*, has been previously introduced [54]. To prevent any misunderstanding, we have chosen a different name for the enzyme originating from *Thermothelomyces thermophilus* examined in this study. Despite our prior publication as *Tt*LPMO9G by Chorozian et al. [30], in this paper, it will be referred to as *Tth*LPMO9G.

*Tth*LPMO9G (GenBank: AEO54509.1) originating from *T. thermophilus*, was modified to construct variants following the instructions provided by the QuikChange site-directed mutagenesis kit. *Tth*LPMO9G variants were constructed by following the instructions of QuikChange site-directed mutagenesis kit (Stratagene, CA, USA), as described elsewhere [55], using pGAP/ttAA9 vector as the template. In the designated experiment, specific primers were used to target certain gene regions. The primer named S28A-F has the nucleotide sequence CACTAACTACAACGCTCCAGTTACTG and is 26 nucleotides long. Its reverse counterpart, S28A-R, shares the same sequence. Additionally, the H140A-F primer has the sequence CAATTGGGTATCGCTAACCCATGGCC, while its reverse counterpart, H140A-R, has the sequence GGCCATGGGTTAGCGATACCCAATTG. Both are also 26 nucleotides in length. The wild-type (WT) *Tth*LPMO9G and its variants were expressed in 500 mL buffered YPD medium under the control of the constitutive glyceraldehyde 3-phosphate dehydrogenase (GAP) promoter for 4 days, as previously described [30]. The enzymes were purified using one-step immobilized metal ion affinity chromatography (IMAC) and the homogeneity of the proteins was corroborated by SDS-PAGE. The concentration of *Tth*LPMO9G protein was quantified using UV spectroscopy at a wavelength of 280 nm. For this measurement, we employed theoretical extinction coefficients, which were calculated using the Expasy ProtParam tool [56]. The computational parameters of the *Tth*LPMO9G protein taken into consideration were: a total of 307 amino acids, a molecular weight of 32,528.56 Da, a theoretical isoelectric point (pI) of 5.95, and an extinction coefficient of 51,965 M^−1^ cm^−1^ [55]. To ensure the loading of copper onto the active site, the enzyme solutions were incubated with a twofold molar excess of CuSO_4_ for 30 min at room temperature. The removal of any remaining free copper was achieved by desalting and final purified enzymes were dialyzed against 20 mM Tris–HCl pH 8 buffer.

### Analysis of oligosaccharide products

The enzyme reaction products were analyzed using HPAEC-PAD. Analysis with HPAEC-PAD was done on a Dionex ICS5000 instrument featuring a CarboPac PA1 guard column (2 by 50 mm) and a CarboPac PA1 column (2 by 250 mm). The use of two mobile phases, A (0.1 M NaOH) and B (1 M sodium acetate in 0.1 M NaOH), was also employed. For the analysis of oxidized products, a 40 min method was employed and the reaction products were eluted at 1 mL/min as described before [51]. Assignment of oxidized oligosaccharides was based on previous literature data [32, 57]. Glucose, xylose, soluble cellooligosaccharides (DP2-6), and xylooligosaccharides (DP2-4) from Megazyme were used as standards. The recording and analysis of chromatograms were carried out using the Chromeleon software. Quantification of the reaction products was based on the standard curve, which was derived from increasing concentrations of standard sugars and the quantification of the areas of the eluted peaks.

### Cellulose binding assay

To investigate the impact of mutations on cellulose binding under non-reductive conditions, a series of experiments were conducted using *Tth*LPMO9G wild type and its mutant variants, based on experimental setup described in [49], incorporating several modifications. This experiment focused on *Tth*LPMO9G variants to bind on PASC. For this purpose, PASC was suspended at a concentration of 1 mg/mL in a sodium acetate, acetic acid buffer (50 mM, pH 5.5), yielding a total volume of 400 μL in 1.5 mL Eppendorf tubes. The binding was initiated by adding *Tth*LPMO9G or variants to a final concentration of 10 μM, followed by incubation at 45°C with stirring at 850 rpm in an Eppendorf Comfort Thermomixer. After a 1-h incubation period, the samples were centrifuged at 15,000×*g* for 10 min, allowing the substrate to settle as residue while the protein content remained in the solution. To evaluate the percentage of bound proteins to the substrate, control samples containing only the enzyme and buffer were prepared. These controls were essential to determine the maximum quantity of protein present in the samples, equivalent to 100% unbound. Additionally, control reactions consisting solely of PASC without any protein content were conducted to monitor the A280 absorption, ensuring that the observed absorbance was specific to protein presence and not influenced by the presence of PASC alone. The protein concentration in each of the samples was subsequently measured using A280 absorption.

### Structure visualization and comparison

Sequence-independent structural comparisons were performed in PyMOL, Schrödinger, Inc. PyMOL, considering all residues but the heteroatoms. *Tth*LPMO9G model structure was derived with AlphaFold, as previously described [30]. The computational site-directed mutagenesis of residues at positions S28A and H140A were accomplished using the mutagenesis wizard in PyMOL. The algorithm implemented in PyMOL generates mutations by replacing the original residue with the desired one, in this case, alanine. Notably, due to alanine’s simple side chain, it offers only one possible rotameric conformation. This inherent limitation of alanine considerably reduces the likelihood of conformational placement errors during the computational mutation process.

### Supplementary Information

**Additional file 1: Figure S1.** presents *Tth*LPMO9G reactions with PASC and ascorbic acid reductant in the presence or absence of catalase. The chromatograms on the left delineate all eluted products, labeled ox-DPA1 to ox-DPA5 for the 13–19 min retention window. The bar chart on the right presents a comparative analysis of eluted products. Control reactions without the enzyme addition consistently resulted in zero area. Bars denote mean values, with error bars indicating the standard error derived from two independent experiments, each performed at least twice. **Figure S2.** Superimposition of ribbon representations of the overall *Tth*LPMO9G structural model (derived from AlphaFold) and the *Phanerochaete chrysosporium* GH61D enzyme (PDB: 4B5Q). The active site is spotlighted within the square. An enlarged depiction of the active site for both enzymes highlights the key amino acids—His149 (H149) for 4B5Q and His140 (H140) for *Tth*LPMO9G. These secondary coordination histidines are demonstrated to occupy identical spatial regions when measurements are taken from the active site His76 for 4B5Q and His70 for *Tth*LPMO9G. The RMSD between the two structures stands at 0.654, comparing the first 201 amino acids between the two proteins. **Figure S3.** SDS-PAGE analysis of purified *Tth*LPMO9G and its variants. The gel presents single bands for the WT *Tth*LPMO9G, as well as for the H140A and S28A variants. All three protein variants exhibit a molecular weight of approximately 55 kDa when compared to the protein ladder. **Figure S4. A** depicts a diagram demonstrating the fluorescence measurements of Amplex® Red fluorometry, which have been converted into H_2_O_2_ concentrations. The oxidase activity of *Tth*LPMO9G 4μΜ variants, as evidenced by the release of H_2_O_2_, is assessed in the presence of 30 μM ascorbic acid. The conversion of the released H_2_O_2_ is illustrated through a standard curve derived from the known concentrations of H_2_O_2_ that were included in the experiment. Different enzyme variants are represented in the figure, each identified by unique symbols. **B** presents a bar chart of V_max_ measurements obtained from the microplate absorbances of 2,6-dimethoxyphenol and H_2_O_2_ in the presence of *Tth*LPMO9G and variants. These measurements are taken until 150 s with a coefficient of determination (R^2^) greater than 0.98. The recorded data reflect the formation of the product coerulignone, with enzymes being diluted and normalized to their initial concentrations. Consequently, the reported units represent the rate of coerulignone generation, quantified as per minute per milligram of enzyme. **Figure S5.** The chromatograms on the left delineate all eluted products, labeled ox-DP_A1_ to ox-DP_A5_ for the 13–19 min retention time frame. The bar chart on the right presents a comparative analysis of each eluted product for the WT and the S28A mutant under the influence of ascorbic acid and both gallic and caffeic acids. Control reactions without the enzyme addition consistently resulted in zero area. Bars denote mean values, with error bars indicating the standard error derived from two independent experiments, each performed at least twice. **Table S1.** Redox Partners for *Tth*LPMO9G—Structures and Classification.

## Data Availability

All data supporting the conclusions of this article are included in the manuscript and its additional files. Samples of materials produced in the current work are available from the corresponding author upon reasonable request.
