# Supplementary material for: Assessing the role of redox partners in TthLPMO9G and its mutants: focus on H2O2 production and interaction with cellulose
Source: Biotechnol Biofuels Bioprod. 2024 Feb 1;17:19. doi: 10.1186/s13068-024-02463-y (PMC10835826; doi:10.1186/s13068-024-02463-y)
Supplement: Supplementary file 1 — Additional file 1: Figure S1. presents TthLPMO9G reactions with PASC and ascorbic acid reductant in the presence or absence of catalase. The chromatograms on the left delineate all eluted products, labeled ox-DPA1 to ox-DPA5 for the 13–19 min retention window. The bar chart on the right presents a comparative analysis of eluted products. Control reactions without the enzyme addition consistently resulted in zero area. Bars denote mean values, with error bars indicating the standard error derived from two independent experiments, each performed at least twice. Figure S2. Superimposition of ribbon representations of the overall TthLPMO9G structural model (derived from AlphaFold) and the Phanerochaete chrysosporium GH61D enzyme (PDB: 4B5Q). The active site is spotlighted within the square. An enlarged depiction of the active site for both enzymes highlights the key amino acids—His149 (H149) for 4B5Q and His140 (H140) for TthLPMO9G. These secondary coordination histidines are demonstrated to occupy identical spatial regions when measurements are taken from the active site His76 for 4B5Q and His70 for TthLPMO9G. The RMSD between the two structures stands at 0.654, comparing the first 201 amino acids between the two proteins. Figure S3. SDS-PAGE analysis of purified TthLPMO9G and its variants. The gel presents single bands for the WT TthLPMO9G, as well as for the H140A and S28A variants. All three protein variants exhibit a molecular weight of approximately 55 kDa when compared to the protein ladder. Figure S4. A depicts a diagram demonstrating the fluorescence measurements of Amplex® Red fluorometry, which have been converted into H2O2 concentrations. The oxidase activity of TthLPMO9G 4μΜ variants, as evidenced by the release of H2O2, is assessed in the presence of 30 μM ascorbic acid. The conversion of the released H2O2 is illustrated through a standard curve derived from the known concentrations of H2O2 that were included in the experiment. Different enzyme variants ar [file 13068_2024_2463_MOESM1_ESM.docx]

**Additional information**

Assessing the Role of Redox Partners in *Tth*LPMO9G and Its Mutants: Focus on H_2_O_2_ Production and Interaction with Cellulose *Koar Chorozian^1^,* *Anthi Karnaouri^3^, Nefeli Georgaki Kondyli^1,2^, Antonis Karantonis^2^, Evangelos Topakas^1,^**

*^1^Industrial Biotechnology & Biocatalysis Group, School of Chemical Engineering, National Technical University of Athens, Zografou Campus, 15772, Greece*

*^2^Laboratory of Physical Chemistry and Applied Electrochemistry, School of Chemical Engineering, National Technical University of Athens, Zografou Campus, 15772, Greece*

*^3^Laboratory of General and Agricultural Microbiology, Department of Crop Science, Agricultural University of Athens, Athens, Greece*

**Corresponding author:*

*Evangelos Topakas, vtopakas@chemeng.ntua.gr, tel.: +30 210 772 3264*


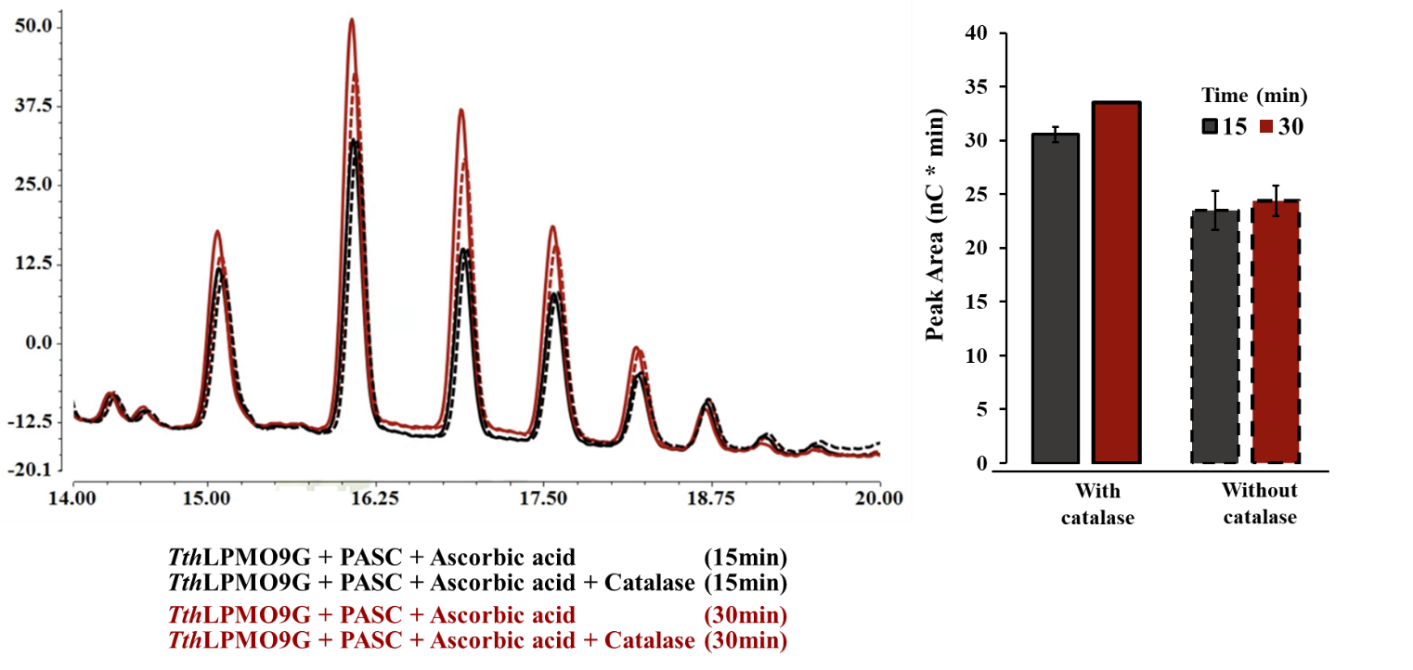


**Figure S1** presents *Tth*LPMO9G reactions with PASC and ascorbic acid reductant in the presence or absence of catalase. The chromatograms on the left delineate all eluted products, labeled ox-DPA1 to ox-DPA5 for the 13-19 min retention window. The bar chart on the right presents a comparative analysis of eluted products. Control reactions without the enzyme addition consistently resulted in zero area. Bars denote mean values, with error bars indicating the standard error derived from two independent experiments, each performed at least twice.

**
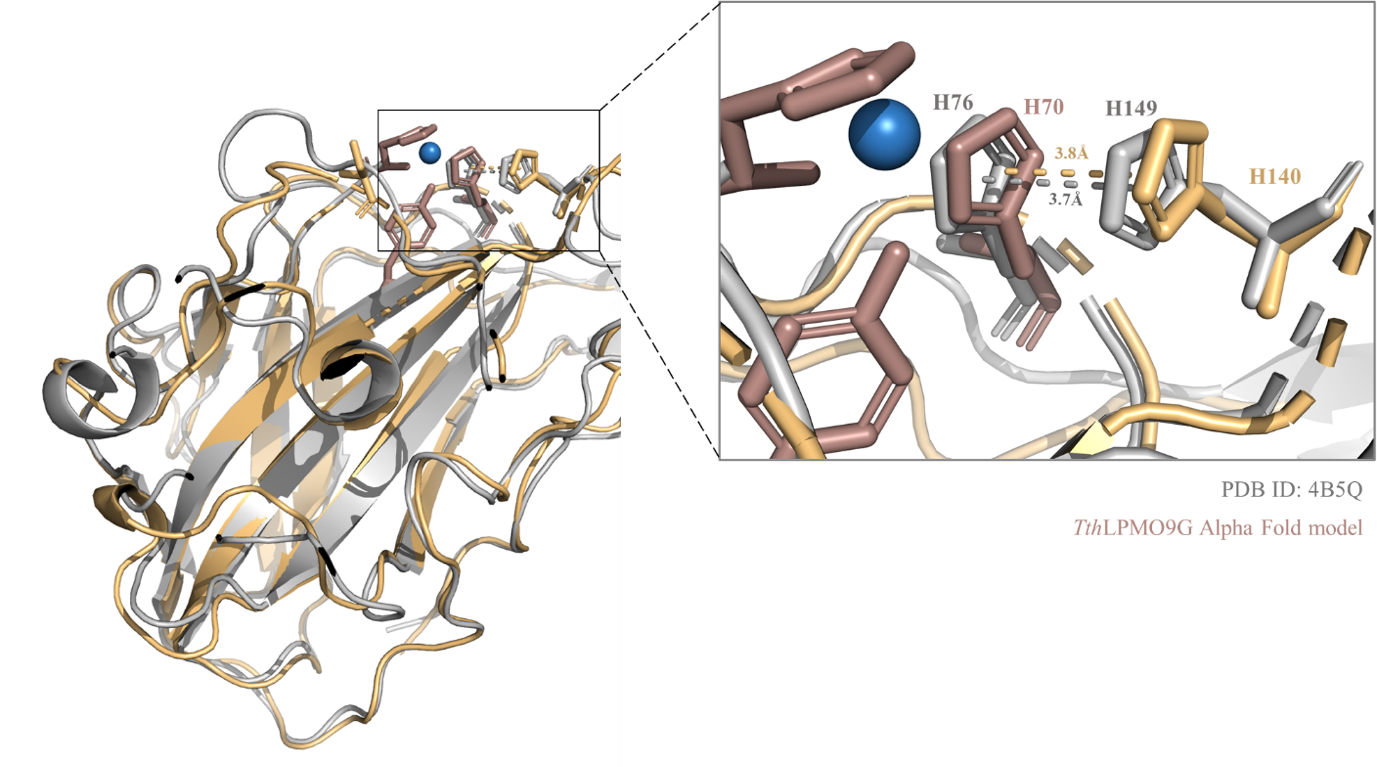
**

**Figure S2.** Superimposition of ribbon representations of the overall *Tth*LPMO9G structural model (derived from AlphaFold) and the *Phanerochaete chrysosporium* GH61D enzyme (PDB: 4B5Q). The active site is spotlighted within the square. An enlarged depiction of the active site for both enzymes highlights the key amino acids - His149 (H149) for 4B5Q and His140 (H140) for *Tth*LPMO9G. These secondary coordination histidines are demonstrated to occupy identical spatial regions when measurements are taken from the active site His76 for 4B5Q and His70 for *Tth*LPMO9G. The RMSD between the two structures stands at 0.654, comparing the first 201 amino acids between the two proteins.


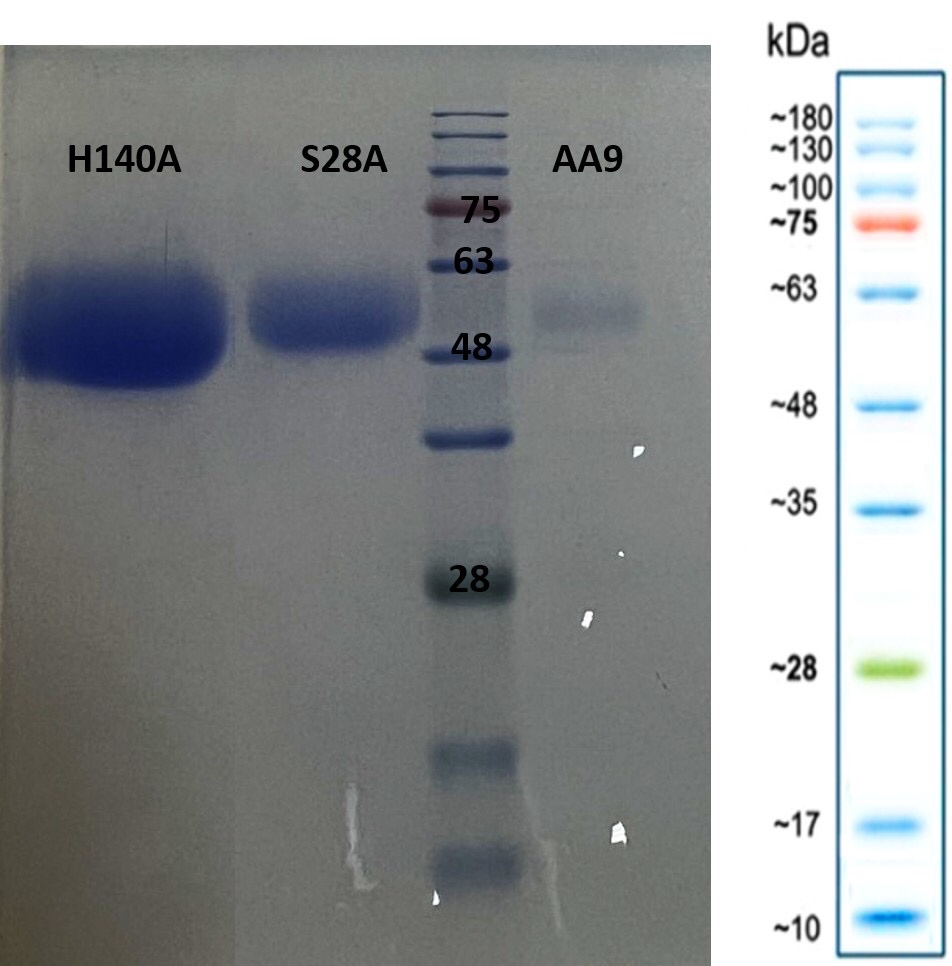


**Figure S3.** SDS-PAGE analysis of purified *Tth*LPMO9G and its variants. The gel presents single bands for the WT *Tth*LPMO9G, as well as for the H140A and S28A variants. All three protein variants exhibit a molecular weight of approximately 55 kDa when compared to the protein ladder.

**A**

**B**


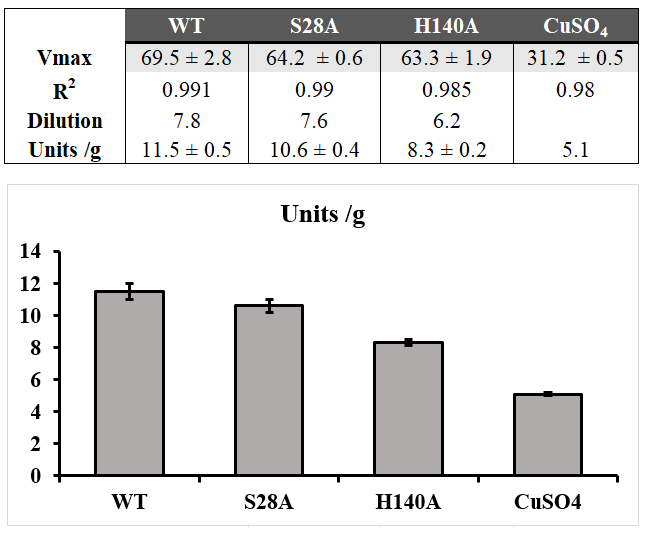


**Figure S4. A** depicts a diagram demonstrating the fluorescence measurements of Amplex^®^ Red fluorometry, which have been converted into H_2_O_2_ concentrations. The oxidase activity of *Tth*LPMO9G 4μΜ variants, as evidenced by the release of H_2_O_2_, is assessed in the presence of 30 μM ascorbic acid. The conversion of the released H_2_O_2_ is illustrated through a standard curve derived from the known concentrations of H_2_O_2_ that were included in the experiment. Different enzyme variants are represented in the figure, each identified by unique symbols. **B** presents a bar chart of V_max_ measurements obtained from the microplate absorbances of 2,6-dimethoxyphenol and H_2_O_2_ in the presence of *Tth*LPMO9G and variants. These measurements are taken until 150 sec with a coefficient of determination (R^2^) greater than 0.98. The recorded data reflects the formation of the product coerulignone, with enzymes being diluted and normalized to their initial concentrations. Consequently, the reported units represent the rate of coerulignone generation, quantified as per minute per milligram of enzyme.

**
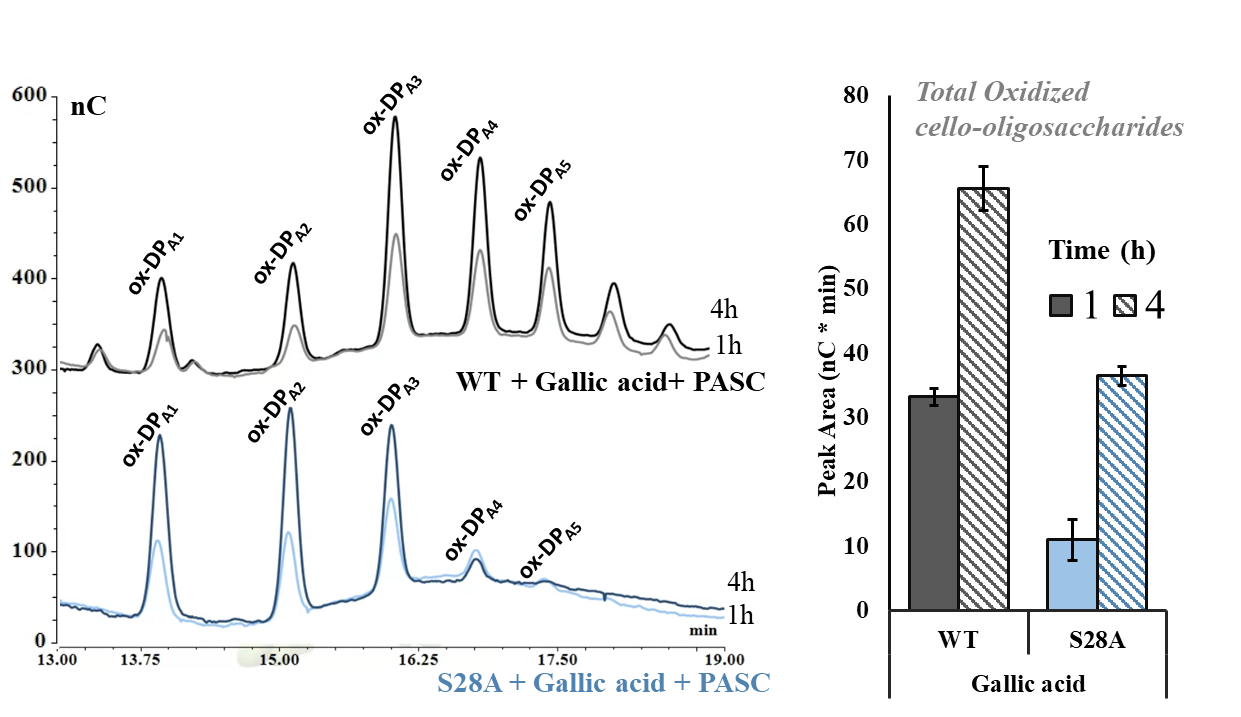
**

**Figure S5.** The chromatograms on the left delineate all eluted products, labeled ox-DP_A1_ to ox-DP_A5_ for the 13-19 min retention time frame. The bar chart on the right presents a comparative analysis of each eluted product for the WT and the S28A mutant under the influence of ascorbic acid and both gallic and caffeic acids. Control reactions without the enzyme addition consistently resulted in zero area. Bars denote mean values, with error bars indicating the standard error derived from two independent experiments, each performed at least twice.

**Table S1.** Redox Partners for *Tth*LPMO9G - Structures and Classification. The table lists various redox partners for *Tth*LPMO9G, providing their names, chemical structures, and classifications. This concise overview facilitates quick reference and comparison of the redox partners associated with *Tth*LPMO9G.

| **Name** | **Structure** | **Chemical classification** |
| --- | --- | --- |
|  |  |  |
| *p*-Coumaric acid | 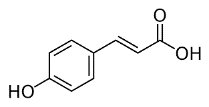   \|  \| \| --- \| | Monophenol |
| Caffeic acid | 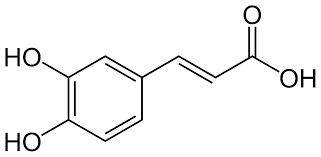 | Dihydroxybenzene |
| Gallic acid | 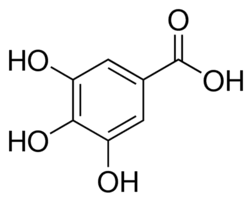 | Trihydroxybenzene |
| Ferulic acid | 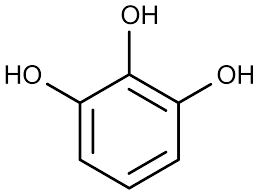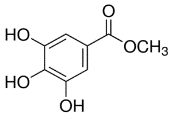 | Methoxyphenol |
| Vanillin | 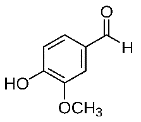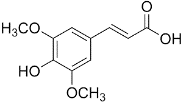 | Methoxyphenol |
| Sinapic acid |  | Methoxyphenol |
| Ascorbic acid | 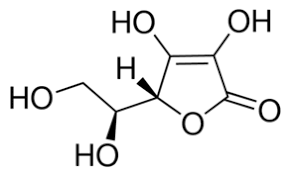 | γ-Lactone |
